# Best practices for the analytical validation of clinical whole-genome sequencing intended for the diagnosis of germline disease

**DOI:** 10.1038/s41525-020-00154-9

**Published:** 2020-10-23

**Authors:** Christian R. Marshall, Shimul Chowdhury, Ryan J. Taft, Mathew S. Lebo, Jillian G. Buchan, Steven M. Harrison, Ross Rowsey, Eric W. Klee, Pengfei Liu, Elizabeth A. Worthey, Vaidehi Jobanputra, David Dimmock, Hutton M. Kearney, David Bick, Shashikant Kulkarni, Stacie L. Taylor, John W. Belmont, Dimitri J. Stavropoulos, Niall J. Lennon, Medical Genome Initiative

**Affiliations:** 1 Department of Paediatric Laboratory Medicine, Genome Diagnostics, The Hospital for Sick Children, Toronto, ON Canada; 2 Rady Children’s Institute for Genomic Medicine, San Diego, CA USA; 3 Illumina Inc., San Diego, CA USA; 4 Laboratory for Molecular Medicine, Partners HealthCare Personalized Medicine, Cambridge, MA USA; 5 Broad Institute of MIT and Harvard, Cambridge, MA USA; 6 Stanford Medicine Clinical Genomics Program, Stanford Health Care, Stanford, CA USA; 7 Department of Laboratory Medicine and Pathology, Mayo Clinic, Rochester, MN USA; 8 Department of Health Sciences Research, Mayo Clinic, Rochester, MN USA; 9 Baylor Genetics and Baylor College of Medicine, Houston, TX USA; 10 HudsonAlpha Institute for Biotechnology, Huntsville, AL USA; 11 Molecular Diagnostics, New York Genome Center, New York, NY USA; 12 Department of Pathology and Cell Biology, Columbia University Irving Medical Center (CUIMC), New York, NY USA; 13 Department of Molecular and Human Genetics, Baylor College of Medicine, Houston, TX USA; 14 Present Address: Department of Laboratory Medicine, University of Washington, Seattle, WA USA; 15 Present Address: Center for Genomic Data Sciences, University of Alabama at Birmingham, Birmingham, AL USA

**Keywords:** Genetic testing, Next-generation sequencing, Laboratory techniques and procedures, Genetic testing

## Abstract

Whole-genome sequencing (WGS) has shown promise in becoming a first-tier diagnostic test for patients with rare genetic disorders; however, standards addressing the definition and deployment practice of a best-in-class test are lacking. To address these gaps, the Medical Genome Initiative, a consortium of leading healthcare and research organizations in the US and Canada, was formed to expand access to high-quality clinical WGS by publishing best practices. Here, we present consensus recommendations on clinical WGS analytical validation for the diagnosis of individuals with suspected germline disease with a focus on test development, upfront considerations for test design, test validation practices, and metrics to monitor test performance. This work also provides insight into the current state of WGS testing at each member institution, including the utilization of reference and other standards across sites. Importantly, members of this initiative strongly believe that clinical WGS is an appropriate first-tier test for patients with rare genetic disorders, and at minimum is ready to replace chromosomal microarray analysis and whole-exome sequencing. The recommendations presented here should reduce the burden on laboratories introducing WGS into clinical practice, and support safe and effective WGS testing for diagnosis of germline disease.

## Introduction

Advances in next-generation sequencing (NGS) over the past decade have transformed genetic testing by increasing diagnostic yield and decreasing the time to reach a diagnosis^1–5^. Targeted NGS multigene panels have come into widespread use and whole-exome sequencing (WES) is a powerful aid in the diagnosis of patients with nonspecific phenotypic features^6–10^ and critically ill neonates^11^, where the differential diagnosis often includes multiple rare genetic disorders^12^. These approaches, however, have both workflow and test content limitations that may constrain their overall efficacy.

Whole-genome sequencing (WGS) can address many of the technical limitations of other enrichment-based NGS approaches, including improved coverage^13,14^, and sensitivity for the detection of structural and complex variants^15^. WGS also enables the identification of noncoding variants, such as pathogenic variations disrupting regulatory regions, noncoding RNAs, and mRNA splicing^16–18^. Emerging uses of WGS include HLA genotyping^19^, pharmacogenetic testing^20^, and generation of polygenic risk scores^21^. Several studies have demonstrated the advantages of WGS for the identification of clinically relevant variants in a wide range of cohorts^22–26^, and have shown the diagnostic superiority of WGS compared with conventional testing in pediatric patients^27–29^ and critically ill infants^30,31^. As a more efficient test, WGS is poised to replace targeted NGS or WES and chromosomal microarray (CMA), as a first-line laboratory approach in the evaluation of children and adults with a suspected genetic disorder^28,32,33^. WGS also has the benefit of periodic reanalysis across multiple variant types, which will increase diagnostic efficacy through updated annotation and analysis techniques^34^.

Although the stage is set for widespread adoption of clinical WGS, technical challenges remain, and standards that address both the definition and the deployment practices of a best-in-class clinical WGS test have not been fully defined. Professional bodies have made progress in providing guidance for clinical WGS test validation^35,36^, and best practices for benchmarking with reference standards and recommended accuracy measures are beginning to emerge^37–39^. It is important to note, however, that these recommendations do not address the specific challenges related to the setup of clinical WGS.

## Scope and methods

To address these challenges, a working group comprised of experts from the Medical Genome Initiative^40^ was created to develop practical recommendations related to the analytical validation of clinical WGS. We decided to focus on the use of a clinical WGS test for the diagnosis of germline disease and that other applications of WGS (such as testing for somatic variants or cell-free circulating DNA) were considered out of scope. As many of the basic principles of laboratory test validation also apply to WGS, this document is not meant to provide a comprehensive description of all the steps of laboratory test validation, but to rather focus on the specific challenges posed by clinical WGS validation.

To identify areas of group consensus and ultimately develop practical recommendations for clinical laboratories, a survey was created that queried working group members on key topics related to analytical validation, including their own current laboratory practices. Biweekly teleconference meetings over a 12-month period were held to share and discuss these current practices, and determine where consensus could be attained. Notably, finding consensus was often difficult due to the variability in validation approaches and the wide range of quality control metrics used among the laboratories. Nonetheless, these recommendations provided herein are meant to aid laboratory personnel who wish to introduce WGS into clinical practice and, more importantly, to support safe and effective WGS testing for diagnosis of germline disease.

## Overview of clinical whole-genome sequencing

**Fig. 1:**
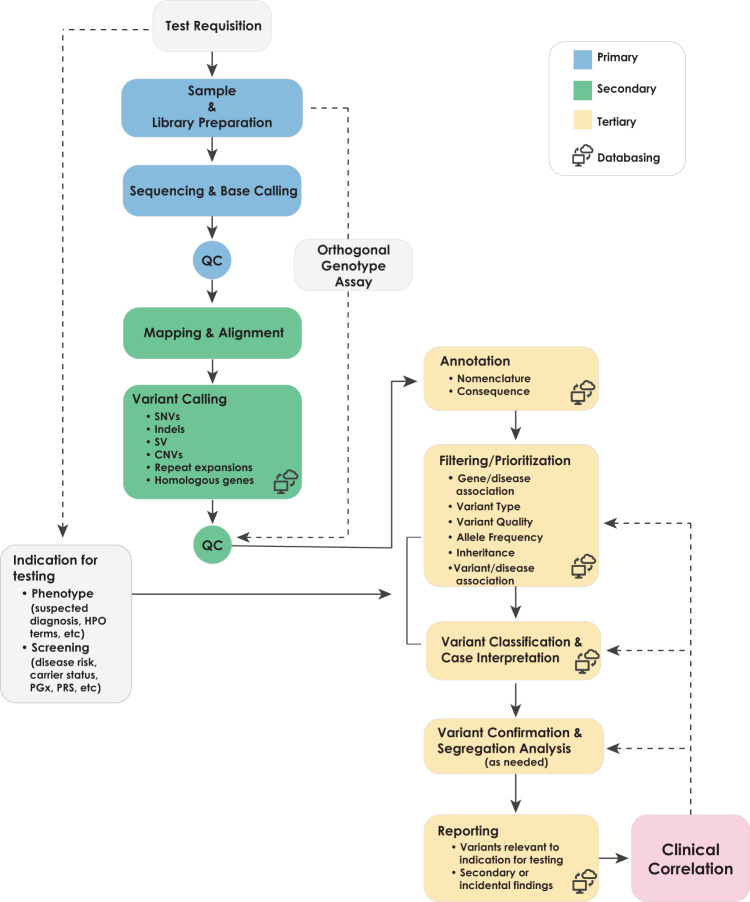
Clinical whole-genome sequencing workflow. The workflow for clinical WGS involves three major analysis steps spanning wet laboratory and informatics processes: primary (blue) analysis refers to the technical production of DNA sequence data from biological samples through the process of converting raw sequencing instrument signals into nucleotides and sequence reads; secondary (green) analysis refers to the identification of DNA variants through read alignment and variant calling; and tertiary (yellow) analysis refers to variant annotation, filtering and prioritization, classification, interpretation, and reporting. Health record information and phenotype can be mined and converted to Human Phenotype Ontology (HPO) terms to aid variant interpretation. Primary analysis involves the sample, and library preparation and sequencing with base calling followed by extensive quality control (QC). During this stage, genotyping with an orthogonal method (SNP-array or targeted assay) is performed for QC purposes. Secondary analysis involves mapping, read alignment, and variant calling. Different classes of variation (SNVs, SV, CNVs, mitochondrial, and repeat expansions) will use different algorithms that can be run in parallel. Aside from QC of alignment and variant calling, the orthogonal genotyping can be used to ensure no sample mix-up has occurred throughout the workflow. Tertiary analysis begins with the annotation of variants followed by filtering, prioritization, and variant classification depending on the phenotype and clinical indication for testing. Classification of variants according to ACMG guidelines may be automated, but the final interpretation involves human intervention and will ultimately be driven by the case phenotype. Variants are reported based on relevance to the primary indication for testing and secondary, or incidental findings not associated with the reason for testing following any necessary confirmation method. Confirmation may be performed with an orthogonal wet laboratory method or in silico examination of the data based on how the test was validated. Clinical correlation (pink) is performed by the ordering physician, which may involve iterative feedback and collaboration with the laboratory (dotted arrows). Throughout the process, collection of aggregate data will be necessary to generate internal allele frequencies and for sharing of interpreted data with repositories.

**Fig. 2:**
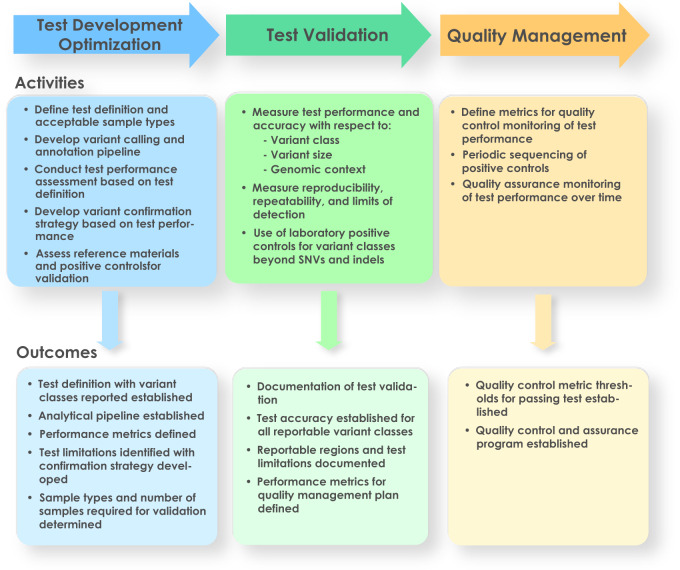
Key steps in the analytical validation of a clinical WGS test. Key steps in the analytical validation of clinical WGS include test development optimization, test validation, and quality management. Each step involves activities that lead to defined outcomes.

All clinical diagnostic testing, including WGS, encompasses the entire process from obtaining a patient specimen to the delivery of a clinical report. The technical and analytical elements of clinical WGS can be separated into three stages: sample preparation, including extraction and library preparation followed by sequence generation (primary); read alignment and variant detection (secondary); and annotation, filtering, prioritization, variant classification, and case interpretation followed by variant confirmation, segregation analysis, and finally reporting (tertiary)^41^ (Fig. 1). These components are common to all high-throughput sequencing tests and informatics pipelines, but differences in components (e.g., informatics algorithms) will result in differences in data quality and accuracy. The focus of this manuscript is the primary and secondary analyses, as these steps relate directly to the evaluation of test performance for the analytical validation of clinical WGS. Elements critical to establishing analytical validity are described below in three sections: (1) test development and optimization, (2) test validation, and (3) ongoing quality management of the test in clinical use. Major steps and activities in the analytical validation are shown in Fig. 2 with key definitions in Box 1. A summary of the key points and recommendations embedded within each of these sections, as well as future considerations can be found in Table 1.

**Table 1:** Summary of key questions and recommendations for the analytical validation of whole-genome sequencing.

	Current state/question	Consensus	Recommendation	Comments and future outlook
Test development and optimization	Test definition considerations			
	What variant classes should be reported in a clinical WGS test?	Yes	• A clinical whole-genome sequencing test should aim to analyze and report on all detectable variant types (Table 1 and Supplementary Fig. 1) • At a minimum, we recommend reporting on SNVs, indels, and CNVs.	• Test definitions are evolving and laboratories should further aim to offer reporting of mitochondrial variants, repeat expansions, some structural variants, and selected clinically relevant genes whose analytical assessment is made difficult by pseudogenes or highly homologous sequence.
	Test performance considerations			
	What comparisons are necessary for WGS to replace other tests such as CMA or WES?	Yes	• Clinical WGS test performance should meet or exceed that of any tests that it is replacing. • Current evidence suggests WGS is analytically sufficient to replace WES and CMA (Table 1, Supplementary Table 1 and Supplementary Fig. 3.	• If clinical WGS is deployed with any established gaps in performance compared to current gold standard tests, it should be noted on the test report. • Robust detection by WGS of some variants is either not equivalent (mosaic SNVs) or has yet to be established (e.g., repeat expansions), but should still be included in the test definition as long as limitations in test sensitivity are defined.
	Which variant types should be confirmed before reporting on a WGS test?	Yes	• The laboratory should have a strategy in place to define which variants need confirmatory testing before reporting. • Until the accuracy of more complex variants (SVs, REs, etc.) is equivalent to currently accepted assays, confirmation with an orthogonal method is necessary before reporting.	• As algorithms improve for complex variant calling and data are acquired to support WGS accuracy compared to currently accepted assays, it is expected that orthogonal confirmation will not be necessary.
	Upfront considerations for test design			
	How should labs define and evaluate high-quality genome coverage and callability?	Some	• Metrics that measure genome completeness should be used to define the performance of clinical WGS, and include overall depth and evenness of coverage. • These should be monitored with respect to callable regions of the genome and the related calling accuracy for each variant type compared to orthogonally investigated truth sets. • An assessment of callable regions should use depth of coverage, base quality, and mapping quality.	• Although consensus was achieved on the concept of the evaluation of genome coverage and callability, consistent methodology, and universal cutoffs could not be established. Expected and suggested values, and ranges are shown in Table 3.
	What reference standards and positive controls should be used for performing clinical WGS validation?	Yes	• Reference standards are useful for the evaluation of calling accuracy across variant type, size, and location. • Analytical validation of clinical WGS should include publicly available reference standards (e.g., NIST and platinum genomes) in addition to commercially available and laboratory-held positive controls for each variant type (see Supplementary Table 3)	• Reference standards are not sufficient on their own for validation and laboratory-held positive controls derived from the same specimen type should also be used.
	How many controls should be used for variant types commonly addressed by the field vs those were standards are still evolving (REs)?	Yes	• For commonly addressed variants like small variants, a low minimal number of controls can be utilized if they include well-accepted reference standards. • For more complex and emerging variant types like RE, a large number of samples are necessary (see Supplementary Table 3).	• As algorithms and reference standard datasets improve, it is expected that fewer samples will be needed for test validation.
Test validation	Performance metrics, variant type, and genomic context			
	What factors should be considered when deciding which metrics to evaluate during test validation?	Some	• The analytical framework should include metrics that account for genome complexity, with special attention to sequence content and variant type.	• Small variants and CNVs have different calling constraints that can be affected differently by low-complexity sequence.
	Which performance metrics should be utilized to ensure the accuracy of clinical WGS?	Some	• Use of GA4GH and FDA recommendations for sensitivity or PPA and precision or TPPV, and lower bound of the 95% CI when truth sets are available. • Repeatability, reproducibility, and limits of detection should be tested. • PPA and NPA against positive controls assessed using a precedent technology is recommended when standard truth sets are not available (see Supplementary Table 1 for example).	• Precision or TPPV is a more useful metric than specificity due to the large number of true negatives expected from clinical WGS.
	What limitations affecting variant calling performance should be considered for a WGS test?	Some	Variant class: • Mosaics: limitation for detection of mosaic variants is low due to low overall read depth. • Structural variants: limitations in the detection of balanced changes (translocation and inversions). • Repeat expansions: limitation in accurately determining expanded sizes. Variant size: • Indels/SV: limitations of calling accuracy for larger indels >20 bp and smaller SVs <1000 bp. Genomic context: • Limitations of accuracy for all variant types in regions of high homology, low complexity, and other technically challenging regions.	• Consensus on limitations for calling performance by variant type was achieved; however, consensus on specific definitions related to variant size and genomic context was not. • Regions that are defined as technically challenging should be documented and made available to those ordering the test.
	Sample number and type for validation			
	How many samples and what types are needed for validation?	Some	• Reference standards are sufficient to assess global accuracy of small variants, but should be supplemented with positive controls. • Beyond small variants, more positive controls are needed; these should reflect the most common pathogenic loci or variants.	• Little consensus on the number of positive controls needed. • Number of samples needed for emerging uses of clinical WGS will evolve as more laboratories validate clinical WGS.
Quality management	Control samples			
	What should be included in ongoing quality control of a clinical WGS test?	Yes	• Identification of a comprehensive set of performance metrics and continual monitoring. • Use of positive controls on a periodic basis.	Less reliance on positive controls for each run; more on the continual monitoring of defined metrics.
	Sequencing quality and performance metrics			
	What sequencing metrics should be monitored for performance?	No	• General agreement in some of the sequencing and analysis metrics that should be used for pass/fail (Table 3). • Unable to reach consensus on which metrics should be used for sample level QC and monitoring, and the corresponding thresholds that need to be met.	Standards for metrics calculations should allow for consensus on thresholds.

Box 1. Definitions of key terms.

**Table:** 

Term	Definition
Analytical validity	A measure of the accuracy with which a test predicts a genetic change.
Callable region (callability)	Regions of the genome where accurate single-nucleotide variant genotype can be reliably derived. Typically expressed as a percentage of non-N reference calls with a passing genotype across a target (whole genome, OMIM genes).
Completeness	Proportion of the genome, or a select region of interest (e.g., exons), that have sufficient, high-quality sequencing reads to enable identification of variants.
Negative percent agreement (NPA)	Equivalent to specificity. The proportion of correct calls in the absence of a variant, reflecting the frequency of false positives (FP). Calculated as the number of true negatives (TN) detected divided by the total number of positions where a variant is absent (TN plus FP).
No-call or invalid call	A position within the testing interval where no variant call can be been made.
Orthogonal confirmation	Verification of a specific variant call using a different testing modality.
Positive percent agreement (PPA)	Equivalent to recall/sensitivity. Ability of the test to correctly identify variants that are present in a sample, reflecting the frequency of false negatives (FN). Calculated as the number of known variants detected (true positives; TP) divided by the total number of known variants tested (TP plus FN).
Precision	Equivalent to TPPV. The fraction of variant calls that match the expected, reflecting the number of FP per test. Calculated as the number of TP divided by the total number of positive calls made (TP plus FP).
Predicted zygosity	In diploid organisms, one allele is inherited from the male parent and one from the female parent. Zygosity is a description of whether those two alleles have identical or different DNA sequences.
Read depth	A measure of the number of sequence reads that are aligned to a specific base or locus.
Repeatability	The percent agreement between the results of successive tests carried out under the same conditions of measurement.
Reproducibility	The percent agreement between the results of tests under a variety (e.g., different operators, machines, and time frames).
Sensitivity or recall	Equivalent to PPA. Ability of the test to correctly identify variants that are present in a sample, reflecting the frequency of FN. Calculated as the number of known variants detected (TP) divided by the total number of known variants tested (TP plus FN).
Specificity	Equivalent to NPA. The proportion of correct calls in the absence of a variant, reflecting the frequency of FP. Calculated as the number of TN detected divided by the total number of positions where a variant is absent (TN plus FP).
Technical positive predictive value (TPPV)	Equivalent to precision. The fraction of variant calls that match the expected, reflecting the number of FP per test. Calculated as the number of TP divided by the total number of positive calls made (TP plus FP).

## Test development and optimization

There are several components of clinical WGS test design that should be taken into consideration as part of test development and optimization. Here, we focus our discussion on some of the unique aspects of clinical WGS, including the test’s definition, test performance comparisons to current methodologies, and upfront considerations for test design. Other components such as sample and library preparation, sequencing methodology, sequence analysis, and annotation are discussed in more detail in the Supplementary Discussion.

### Test definition considerations

Analytical validation requirements will vary based on test definition, which includes both technical considerations and the intended use in a patient population. Although clinical WGS may be used for multiple indications (e.g., inherited disorders, cancer, and healthy individuals), this document focuses on using clinical WGS for individuals with a suspected monogenic germline disorder as the primary use case. The principles of analytical validity described here, however, are applicable to all uses of clinical WGS.

Establishing a test definition for clinical WGS designed to diagnose germline disorders can be challenging for laboratories due to the complexity of the test. Clinical WGS tests are predicated on a specific test definition that delineates both the variant types to be reported and the regions of the genome that will be interrogated (including any limitations), which may vary depending on the variant type. The challenge, due to the comprehensive nature of WGS variant detection, is whether the test definition should be agnostic to phenotype and based on the classes of variants detected or defined for a specific phenotype, since specific loci can be interrogated and reported. The most effective use of genome sequencing at this time is in the evaluation of clinical presentations with a broad range of potential genetic etiologies. However, since it is possible to interrogate specific loci and associated variant types with clinical WGS (e.g., *SMN1* deletions for SMA or *FMR1* expansions for Fragile X), test definitions will broaden in scope and evolve as analytical performance improves.

Classes of clinically relevant genetic variation detectable by clinical WGS are summarized in Table 2, and include single-nucleotide variants (SNVs), small deletions, duplications, insertions (indels), structural variation (SV), including copy number variation (CNV) and balanced rearrangements, mitochondrial (MT) variants, and repeat expansions (REs)^15^. The accuracy of detection for some of these variant classes is well established, whereas other classes are technically possible but data demonstrating sufficient detection accuracy are still emerging. A clinical WGS test should aim, wherever possible, to analyze and report on all possible detectable variant types. We recommend SNVs, indels, and CNVs as a viable minimally appropriate set of variants for a WGS test. Laboratories should further aim to offer reporting of MT variants, REs, some structural variants, and selected clinically relevant genes whose analytical assessment is made difficult by pseudogenes or highly homologous sequence (Table 1 and Supplementary Fig. 1). We note that laboratories may not be able to validate all classes of variation prior to initial launch of clinical WGS, and that a phased approach to validation and subsequent test offering may be necessary. Ultimately, the laboratory must provide clear test definitions and identify factors affecting reportable variant types to ordering physicians. For example, if using a specimen source expected to yield limited DNA quantity, PCR for library preparation may be required, and reporting of CNVs^42^ and REs^43^ will be adversely affected.

**Table 2:** Variant types detectable and reportable from clinical WGS.

Variant type	Gene(s) (if applicable)	Disorder(s)	References (if applicable)
SNVs and small insertions and deletions^a^ (1–50 base pairs)	N/A	Heritable disease	Zook et al.^37^ Eberle et al.^58^
Copy number variation^a^ (deletions and duplications)	N/A	Heritable disease including known microdeletion/duplication syndromes	Gross et al.^33^ Stavropoulos et al.^27^ Lindstrand et al.^45^
Mitochondrial variation^b^ (SNVs, deletions, duplications, and heteroplasmy of at least 5%)	N/A	Known mitochondrial disorders	Duan et al.^56^
Structural variants^b^	N/A	Heritable disease including those caused by translocations, inversions, and other genomic rearrangements	Lindstrand et al.^45^
Repeat expansions^c^	*FMR1*	Fragile X and related disorders	Dolzhenko et al.^43^
	*HTT*	Huntington disease	
	*SCA1*	Spinocerebellar ataxia 1	
	*DMPK*	Myotonic dystrophy 1	
	*C9orf72*	Amyotrophic lateral sclerosis	
Selected pseudogenes^c^	*SMN1 and SMN2*	Spinal muscular atrophy	Chen et al.^57^
	*CYP21A2*	21-Hydroxylase deficiency	
	*CYP2D6*	Codeine sensitivity	
	*HBA1 and HBA2*	Alpha thalassemia	
	*PMS2*	Colorectal cancer	
	*PKD1*	Polycystic kidney disease 1	

^a^Recommended minimum variant types for clinical validation of WGS. Copy number variation is defined here as unbalanced changes (deletions and duplications) that are at the resolution of chromosomal microarray analysis.

^b^Some initiative groups have clinically validated. Structural variants are defined here as any genomic alteration >50 base pairs, including balanced and unbalanced changes.

^c^Examples of targeted loci that could be validated and reported as part of a clinical WGS test.

### Test performance considerations

Regardless of the variant types a laboratory may choose to report, a thorough performance comparison between the WGS test and any current testing methodology is warranted to demonstrate that the analytical performance is sufficient for clinical use. Clinical WGS test performance should aim to meet or exceed that of any tests that it is replacing. If clinical WGS is deployed with any established gaps in performance compared to current reference standard tests, it should be noted on the test report (see Table 1). The most immediate and obvious use of clinical WGS is replacement of genome-wide tests, such as WES and CMA. WGS has been shown to be analytically superior to WES for the detection of variants affecting protein function^32,44^, and there is emerging evidence that the analytical detection of CNVs from WGS is at least equivalent to CMA^27,33,45^ (Supplementary Table 1).

For the detection of some variant types, it is important to acknowledge that clinical WGS may not be equivalent to current methods and that robust detection has yet to be established. For example, detection of low-level mosaicism represents an important limitation of clinical WGS (at 40× mean depth) compared to WES or targeted panels, where loss of performance may be a significant issue for some indications (e.g., epileptic encephalopathy)^46^. As previously mentioned, although more complex variant types like those mentioned above (e.g., MT variants with varying levels of heteroplasmy, REs, etc.) can be identified using WGS, we recognize that in some cases the detection accuracy of these variant types may not yet be equivalent to currently accepted assays. There is still inherent value to including these variant classes in the test definition of clinical WGS to ensure as complete a test as possible, as long as limitations in test sensitivity are clearly defined. As with any genetic assay, the test definition should clearly state that a negative report in these instances does not preclude a diagnosis. Laboratories planning to report on complex variant types must include the test limitations in the report, and have a detailed confirmatory test strategy in place. It is consensus of this Initiative that confirmatory testing of these variant types using an orthogonal method is necessary before reporting (Table 1).

### Upfront considerations for test design

Upfront considerations for WGS test design, such as sample and library preparation, sequencing methodology, sequence analysis, and annotation generally follow current guidelines^35,36,47^ and are discussed in the Supplementary Discussion. More complex test design considerations that are specific to clinical WGS, such as evaluation of metrics to determine suitable WGS test coverage, and the number and type of samples necessary for validation are discussed below.

### Evaluation of genome coverage, completeness, and callability

Defining and evaluating high-quality genome coverage is one of the most important considerations in clinical WGS test development, since it directly relates to the amount of data required to accurately identify variants of interest. Metrics that measure genome completeness should be used to define the performance of clinical WGS, and include overall depth and evenness of coverage. These measures should be monitored with respect to callable regions of the genome and related calling accuracy for each variant type compared to orthogonally investigated truth sets (Table 1). While universal cutoffs are not yet established, a combination of depth of coverage, base quality, and mapping quality is recommended to assess callability^48^. The majority of laboratories in this initiative calculate both raw and usable coverage, the latter metric relating to reads used in variant detection and excluding poorly mapped reads, low-quality base pairs, and overlapping paired reads. All sites have evaluated the performance of clinical WGS, using varying mean depth of coverage, and assessed the completeness and accuracy of variant calling in specific target files, such as a reference standard, or comparison to the method clinical WGS is replacing (e.g., WES; Supplementary Figs. 2, 3). Variability in assessment methodology can result in differences in metrics and cutoffs (Table 3); however, when genome completeness was assessed across three of the sites in this initiative using reference standards the values ranged from 97.7–98.1%, suggesting some consistency in sequencing genomes across laboratories (Supplementary Table 2). If the laboratory is providing WGS from different DNA sources, these evaluations should be completed for each specimen type.

**Table 3:** Metrics for clinical whole-genome sequencing.

Metric	Description	Type (threshold) or typical expected value
Examples of pass/fail metrics		
Sample identity	Concordance with genotype (orthogonal and/or family structure when available).	Pass/fail (match)
Contamination^a^	The estimated level of sample cross-individual contamination based on a genotype-free estimation.	Pass/fail (≤2%)
Gb ≥ Q30^b^	Total aligned gigabases (Gb) of data with base quality score >Q30.	Pass/fail (≥80 Gb)
Autosome mean coverage^c^	The mean coverage across human autosomes, after all filters are applied.	Pass/fail (≥30)
% Callability^d^	Percent of non-N reference positions in autosomal chromosomes with a passing genotype call.	Pass/fail (>95%)
Examples of metrics to monitor		
%Q30 bases total	The percentage of bases that meet Q30 scores.	≥85%
20×%^e^	The fraction of non-N autosome bases that attained at least 20× sequence coverage in post-filtering bases.	≥90%
PF reads aligned %	The percentage of passing filter (PF) reads that align to the reference sequence.	>98%
PF aligned Q20 bases^f^	The number of bases aligned to the reference sequence in PF reads that were mapped at high quality and where the base call quality was Q20 or higher.	>1.0E + 11
Adapter-dimer %	The fraction of PF reads that are unaligned and match to a known adapter sequence right from the start of the read.	<0.2%
Chimera %	The percentage of reads that map outside of a maximum insert size (usually 100 kb) or that have the two ends mapping to different chromosomes.	<1%
Duplication %	The percentage of mapped sequence that is marked as duplicate.	<10%
Median insert size^g^	The median insert size of all paired end reads where both ends mapped to the same chromosome.	>300 bp
Excluded total %	The percentage of aligned bases excluded due to all filters.	<15%

^a^Laboratories in the Medical Genome Initiative use a threshold of <1% for germline WGS from peripheral blood.

^b^Some laboratories in the Medical Genome Initiative use a similar metric of ≥85 Gb unaligned Q30 sequence.

^c^Laboratories in the Medical Genome Initiative use either 30× or 40× mean coverage as a cutoff.

^d^Callability or the fraction of the genome where accurate calls can be made can be calculated in different ways. The description in the table represents one way to calculate callability, but there are others including using the percentage of base pairs that reach a read depth (RD) of 20, with base quality (BQ) and mapping quality (MQ) of 20.

^e^Measure of completeness. Depth of coverage at 15× also used by some laboratories along with a mapping quality cutoff (>10). Targets will also vary; laboratories may measure across genome, exome, OMIM morbid map genes, and positions or exons with known pathogenic regions.

^f^Some laboratories in the Medical Genome Initiative use Q10 Bases.

^g^Mean insert size may also be used. The mean insert size is the “core” of the distribution. Artifactual outliers in the distribution often cause calculation of nonsensical mean and standard deviation values. To avoid this, the distribution is first trimmed to a “core” distribution of ±*N* median absolute deviations around the median insert size. By default, *N* = 10, but this is configurable.

### Reference standard materials and positive controls

High-quality reference standard materials and positive controls with associated truth datasets are a necessary resource for laboratories offering clinical WGS. The analytical validation of clinical WGS should include publicly available reference standards in addition to commercially available and laboratory-held positive controls for each variant type. For variant types commonly addressed by the field, including SNVs and indels, a low minimal number of controls can be utilized if these include well-accepted reference standards. For variant types where standards are still evolving (e.g., REs), a larger number of samples should be employed (Table 1). The National Institute of Standards and Technology (NIST) NA12878 genome and Platinum Genomes are routinely utilized by NGS laboratories seeking to establish WGS analytical validity^47^. These genomes have the benefit of thousands of variants that have been curated and confirmed across many technologies^49,50^. Within this initiative, all groups have used NA12878 for validation, and most groups also utilize the Ashkenazi Jewish and Chinese ancestry trios from the Personal Genome Project that are available, as reference materials with variant benchmarks^37^ (Supplementary Table 3).

The ability to subcategorize analytical performance by variant type is another benefit of using well-characterized reference materials. Genome-wide estimates of sensitivity often mask poor performance in certain sequence contexts or across different variant attributes. For example, the sensitivity of large indel detection (>10 bp) in regions of high homology will be poorer compared to detection of smaller indels in less complex regions. Understanding performance in difficult regions of the genome is important for accurately representing the limitations of the assay, and setting benchmarks against which new analytical tools and methods can be developed. The Global Alliance for Genomics and Health (GA4GH) Benchmarking Team recently developed tools (https://github.com/ga4gh/benchmarking-tools) to evaluate performance in this way. Currently, all members of this initiative have incorporated or intend to use the results of such an analysis in their analytical validation study.

Reference standard materials alone are not sufficient to establish validity of a test, however. For example, both the specimen and disease context must also be taken into consideration when sourcing samples for a validation study. For clinical WGS laboratories in this consortium, specimen context has included determination of the acceptable sample types (e.g., blood, saliva, and tissue) with associated representative positive controls. Some pathogenic variants, including short tandem repeats, low copy repeats, SVs with breakpoints within nonunique sequences, paralogs, and pseudogenes, occur in regions of the genome that are difficult to sequence, align, and map. If analysis and reporting of these loci is planned, the laboratory should perform validation assessments on samples with these specific variant types to determine robustness. Since performance expectations may not be well established for these variants, a large number of positive controls should be used (see below and Supplementary Table 3).

## Test validation

Clinical WGS requires a multifaceted approach to analytical validation due to the large number of rare genetic disease loci, the number and different classes of variation that can be detected, and the genomic context-driven variability in variant calling accuracy. Traditional summary statistics defining performance metrics across the entire assay are necessary, but not sufficient. The analytical validation framework should include metrics that account for genome complexity, with special attention to sequence content and variant type (Table 1). For example, sequence level and copy number variants have different calling constraints that can be affected differently by low-complexity sequence. Specific test validation recommendations that address these and other clinical WGS-specific validation requirements are discussed in detail below. Other considerations that are not unique to clinical WGS include sequencing bias, repeatability and reproducibility, limits of detection, interference, and regions of homology are discussed in the Supplementary Discussion along with disease-specific variant validation (e.g., SMA testing), software validation, and test modification and updates.

### Performance metrics, variant type, and genomic context

Analytical validation is the first step in ensuring diagnostic accuracy and is classically measured in terms of sensitivity (recall) and specificity. However, this initiative agrees with current recommendations from the GA4GH to use precision as a more useful metric than specificity, owing to the large number of true negatives expected by clinical WGS^38^. The FDA suggests similar, albeit slightly different metrics, for validation of NGS assays, including positive percent agreement (PPA; sensitivity), negative percent agreement (NPA; specificity), and technical positive predictive value (TPPV; equivalent to precision above), as well as reporting the lower bound of the 95% confidence interval (CI)^39^. Relevant definitions and calculations are provided in Box 1.

This initiative recommends following published guidelines as described above, as the performance metrics are generally applicable to clinical WGS. In addition to the global metrics of accuracy (sensitivity, precision), repeatability (technical replicates performed under identical conditions), reproducibility (comparison of results across instruments), and limits of detection assessment (e.g., mosaic SNVs) should also be measured. For SNV and indels, gold standard reference data are available, as described above and can be used to calculate performance metrics^47^. Other variant types may not have standard truth sets available, so comparative metrics should be confined to PPA and NPA against laboratory or commercially acquired samples assessed, using a precedent technology. Laboratories may also consider creating virtual datasets and analytically mixed specimens for validation of the variant types that may not have standard truth sets available.

Performance thresholds should be predetermined and matched to clinical requirements for low diagnostic error rates. Flexibility in performance thresholds at the stage of variant calling may be acceptable, as long as these deviations are documented and laboratory procedures include additional confirmatory assessments. These can include additional bioinformatics analyses, manual inspection by analysts, and orthogonal laboratory testing. The amount of data being examined in a clinical WGS test requires that confirmatory methods be restricted to small subsets of the data with potentially high clinical impact. No calls and invalid calls should not be used in calculations of sensitivity, precision, or TPPV in the validation of variant calling. Instead, these should be documented separately as part of the accuracy of the test and, where possible, genomic intervals that routinely have low map quality and coverage should be flagged in the clinical WGS test definition.

Identification of different variant types require unique calling algorithms, resulting in differences in analytical performance. Further stratification by size is warranted for some common variant types to provide greater insight into overall test performance. For example, GA4GH recommends binning insertions, deletions, and duplications into size bins of <50, 50–200, and >200 bp (ref. ^51^), although it is important to note that most laboratories in this initiative assess additional smaller bins (Supplementary Fig. 4). For CNVs, size bins, and minimum cutoffs are similar to the maximum resolution of current clinical CMA, which can vary from 20 to 100 kb, depending on the platform used. Laboratories in this initiative that currently offer CNVs as part of the test report events at the resolution of CMA using a depth-based CNV caller, whereas smaller CNV events often require split or anomalous read pair information partnered with a depth assessment^52^.

Variant calling performance can be affected by the sequence context of the region itself, or, in the case of large variants, the surrounding bases. Currently, there are no best practices for the identification of systematically problematic regions or comprehensive population-level truth datasets, but all members of this initiative have developed internal methods to identify such regions. These include regions where clinical WGS may perform poorly, including paralogous genes, which are excluded from the test definition in order to guide appropriate clinical ordering. The initiative also recommends that regions identified as systematically problematic, or that negatively affect variant calling tied to particular variant types, are documented as part of the test validation and made available to ordering clinicians upon request. Some resources already exist for annotation of genes with high homology, and can be used as a starting point (https://www.ncbi.nlm.nih.gov/books/NBK535152/). Limitations affecting variant calling performance observed during validation should be clearly stated on the report and should include reference to variant types, sizes, and genomic context (Table 1).

### Sample number and type for validation

The number of samples and specimen types required for clinical WGS validation links back to the test definition and the variant types or known disease loci that the laboratory intends to report. It is not technically or practically feasible to validate all possible pathogenic variants genome wide. Thus, we recommend that the number of samples required for validation be guided by variant type or the targeted locus being interrogated. For small variants (SNVs and indels), members of this initiative agree that the repeatable and accurate assessment of genome reference standards is sufficient to establish global accuracy, but this should be supplemented with patient positive controls containing a range of clinically relevant variants. Interestingly, the number of positive controls used by laboratories in this consortium for small variants varied between 10 and 85 (Supplementary Table 3), reflecting a broad range of practice amongst laboratories.

Validation of variant types beyond small variants require increased numbers of positive controls, and should include the most commonly affected genes, loci, or pathogenic variants if targeting a specific locus. The number of specific variants that should be assessed may vary according to variant type, genomic context, and the availability of appropriate reference samples. Where possible adhering to a statistically rigorous approach similar to that outlined by Jennings et al.^53^, which incorporates a confidence level of detection and required probability of detection, is recommended. When applying this method and requiring a 95% reliability with 95% CI, at least 59 variants should be used in the performance assessment, as has been previously published^36,53^. Taking CNV validation as an example, members of this initiative have used between 7 and 42 positive controls (Supplementary Table 3), and included common microdeletion and duplication syndromes (Supplementary Table 1). For other emerging uses of WGS that specifically target loci (e.g., targeted RE or SMN1), many more positive and negative controls are necessary to assess accuracy. As test scope continues to broaden, we expect consensus to emerge on the recommendation of the number of controls required for validation based on the experience of this initiative and others in the community.

## Quality management

As with any laboratory test, groups performing clinical WGS should have a robust quality management program in place for quality control and quality assurance, following applicable regulatory guidance from CLIA (www.cdc.gov/clia), CAP (https://www.cap.org/), and ISO (www.iso.org). Much of the guidance from these regulatory bodies is broadly applicable to any laboratory test, including clinical WGS, and is not discussed here. Rather, we touch on a few points to consider for clinical WGS test quality management focusing on control samples, sample identity, library preparation, sequencing quality and performance metrics, and bioinformatics quality assurance. A list of sequencing and performance metrics examples (many of which are discussed in the following sections) can be found in Table 3. This table features a brief description of each metric, as well as suggested cutoffs or ranges for metrics considered pass/fail and those that should be monitored.

### Control samples

One of the biggest challenges for laboratories offering clinical WGS is the application of controls to comply with regulatory guidelines. Guidelines recommend the use of positive, negative, and sensitivity controls (e.g., CAP Molecular Pathology Checklist, August 2018—MOL.34229 Controls Qualitative Assays) to ensure that all steps of the assay are successfully executed without contamination. Ongoing quality control of a clinical whole-genome test should include identification of a comprehensive set of performance metrics, continual monitoring of these metrics across samples over time, and the use of positive controls on a periodic basis dependent on overall sample volume (Table 1). Although the inclusion of a control reference standard in every sequencing run is ideal, it is not practical or financially viable for a laboratory performing clinical WGS. Moreover, the use of positive and negative controls may be informative for the overall performance of a sequencing run, but will not be reflective of sample-specific differences and may incorrectly indicate adequate test performance.

There are additional positive and negative control strategies that some laboratories may choose to employ. Some of the groups in the initiative use PhiX, which represents the empirical measure of sequencing error rate. For variant positive controls, one approach is the use of low-level spike-ins of well-characterized positive control samples that include a spectrum of variants in each sequencing run. Similarly, some groups in the initiative are exploring the use of synthetic spike-in constructs, including Sequins^54^, which can be added to a run at a low level (<1% of reads) and enable a performance assessment that can serve as a process control for at least some variant types. Within this initiative, most groups run a reference standard at periodic intervals, and check for deviations from expected calling accuracy and concordance with previously run samples.

### Sample identity

Given the multistep processes needed to generate a final result, a sample identity tracking procedure within the laboratory is recommended during tube and instrument transfers, and to confirm the integrity of the final results. Implementing this tracking procedure will mitigate the risk of sample mix-up through the analytical steps of the assay, but will not necessarily detect other pre-analytical issues, such as labeling or sample collection errors. Although there is no standard method employed by initiative members, examples of sample tracking include comparison of WGS data to SNPs genotyped with a multiplex assay or custom microarray, STR marker analysis, or spike-in methodology. Regardless of sample tracking method, discordance in genotype between WGS and orthogonal testing data results in failure of the test (Table 3). Methods similar to those described above should be used when case-parent trios are sequenced, or when other family members are included in the clinical testing strategy. Formal checks for Mendelian errors to establish parentage and to assess relatedness among other family members should be computed using standard methods.

### Library preparation

The yield and quality (e.g., fluorometry and size range) of the DNA should have defined criteria for acceptance that allows a DNA sample to be passed to library preparation and sequencing. For clinical WGS, sample pooling and molecular barcoding is utilized in the majority of laboratories. Some platforms benefit from a dual-barcoding strategy (i.e., a barcode on each end of the library molecule) to reduce the possibility of barcode hopping on the flowcell^55^. Quality metrics (e.g., library concentration) with acceptance thresholds must be established and the results from each sample must be documented. For sample and library preparation, procedures are needed to detect and interpret systematic drops in quality and/or the percentage of samples meeting minimum quality requirements. A control for library preparation may be used to monitor quality, and troubleshoot preparation versus sample issues and a non-template control can be used to monitor systematic contamination.

### Sequencing quality and performance metrics

Test run quality metrics and performance thresholds for clinical WGS should be assessed at the sample level as part of quality control. A quality assurance program should periodically monitor quality metrics over time and identify trends in test performance related to reagent quality, equipment performance, and technical staff.

Clinical WGS sample level quality metrics describe whether the biological specimen and end-to-end test are technically adequate (i.e., whether the test provides the expected analytical sensitivity and technical positive predictive value for all variant types (SNVs, indels, CNVs, and SVs)) within the reportable range of the genome established during test validation.

Quality metrics are calculated for every run of the instrument, and after alignment and variant calling (see Supplementary Discussion for expanded description). Test development optimization and validation processes establish which metrics are reviewed for every sample, but it can be challenging for laboratories to determine appropriate thresholds. Examples of sequencing quality and performance metrics used by members of this consortium to evaluate WGS for pass/fail and monitoring are listed in Table 3. Important metrics for passing samples include the total gigabases (Gb; >Q30) produced per sample, the alignment rate of purity-filtered bases (PF reads aligned %), the predicted usable coverage of the genome (mean autosomal coverage), proportion of reads that are duplicates (% duplication), the % callability (positions with passing genotype call), and any evidence of sample contamination (% contamination). For clinical WGS, it is particularly important to monitor global mapping metrics and assess clinically significant loci for completeness (e.g., OMIM genes and ClinVar pathogenic variants).

Mean coverage and completeness of coverage are commonly used metrics, but as discussed previously, these may be calculated differently across groups (see previous section on coverage evaluation). It is important to note that at the time of publication, the initiative was unable reach a high level of consensus as to which metrics should be used and the corresponding thresholds that need to be met to qualify, as a passing clinical WGS test. There was general agreement on the types of measures that are important (Table 3), but often these were calculated in different ways, which made reaching consensus difficult. This is likely a reflection of the evolving technology and the way in which each group validated testing in the absence of accepted guidelines. More data and laboratory experience are needed before consensus on performance metrics thresholds that define a clinical WGS test can be established.

### Bioinformatics quality assurance

Clinical bioinformatics pipelines developed for the analysis of clinical WGS tests are complex, and require a robust quality assurance program for both ongoing monitoring of metrics and pipeline updates^36^. Due to the continual updating of software versions (e.g., read aligners and variant callers) and data sources for annotation (e.g., OMIM, Clinvar, etc.) the development, validation, and deployment cycles can be challenging for laboratories. Pipeline versions need to be revalidated when updated (see Supplementary Discussion “Software validation”) and a system to track versions with parameters and implementation date must be employed. All code changes need to be documented along with versions of data sources. Pipelines can be tested with reference standards to ensure that they are reproducible and complete without errors.

### Summary

Clinical WGS is poised to become a first-tier test for the diagnosis of those individuals with suspected genetic disease. Although some guidelines are beginning to emerge that offer recommendations for the analytical validation of genome testing, specific challenges related to the setup, and deployment of clinical WGS are not addressed. In this document, we aimed to address these gaps through consensus recommendations for the analytical validation of clinical WGS, based on the experiences of members of the Medical Genome Initiative. We focused on providing practical advice for test development optimization, validation practices, and ongoing quality management for the deployment of clinical WGS. Even amongst members within the initiative, it was challenging to come to a consensus on specific recommendations since there are often different, but equally valid approaches to the analytical validation of WGS. Another reason for the lack of consensus is the rapid advancement of the field; the process of WGS is continually being updated and improved meaning laboratories are often at different stages of implementation. However, members of this initiative agreed upon the endorsement of clinical WGS as a viable first-tier test for individuals with rare disorders, and that it should replace CMA and WES.

The recommendations provided here are meant to represent a snapshot of the current state of the field, and we expect best practices to continue to evolve. Although reaching consensus on specific validation-related practice was not always possible, a sentiment shared by all groups was that establishing standards in clinical WGS is difficult but critically important. Collaborative efforts and communication both within, and among research and healthcare institutions are essential to establishing guidelines and standards to increase access to high-quality clinical WGS, while minimizing patient risk. It is clear that much work is needed in the community to establish clear consensus around many of the analytical principles that define a valid clinical genome test.

To this end, our group is committed to providing best practices on clinical WGS topics both upstream and downstream from analytical validity, including genome interpretation, data infrastructure, and clinical utility measures.

## Supplementary information

Supplementary Information

## Data Availability

The data analyzed during the current study are available from the corresponding author on reasonable request.
