# Supplementary material for: Best practices for the analytical validation of clinical whole-genome sequencing intended for the diagnosis of germline disease
Source: NPJ Genom Med. 2020 Oct 23;5:47. doi: 10.1038/s41525-020-00154-9 (PMC7585436; doi:10.1038/s41525-020-00154-9)
Supplement: Supplementary file 1 — Supplementary Information [file 41525_2020_154_MOESM1_ESM.pdf]

SUPPLEMENTARY INFORMATION

MEDCIAL GENOME INITIATIVE MEMBERS

Christian R. Marshall[1*], Shimul Chowdhury[2], Ryan J. Taft[3], Mathew S. Lebo[4,5], Jillian G. Buchan[6,#], Steven M. Harrison[4,5], Ross Rowsey[7], Eric W. Klee[7,8], Pengfei Liu[9], Elizabeth A. Worthey[10,%], Vaidehi Jobanputra[11,13], David Dimmock[2], Hutton M. Kearney[7], David Bick[10], Shashikant Kulkarni,[9,12] Stacie L. Taylor[3], John W. Belmont[3], Dimitri J. Stavropoulos[1], Niall J. Lennon[5], Heidi Rehm[5], Euan Ashley[6]

SUPPLEMENTARY FIGURES

Supplementary Figure 1: Analytical Validation of Variant Categories Across Medical Genome Initiative Sites.

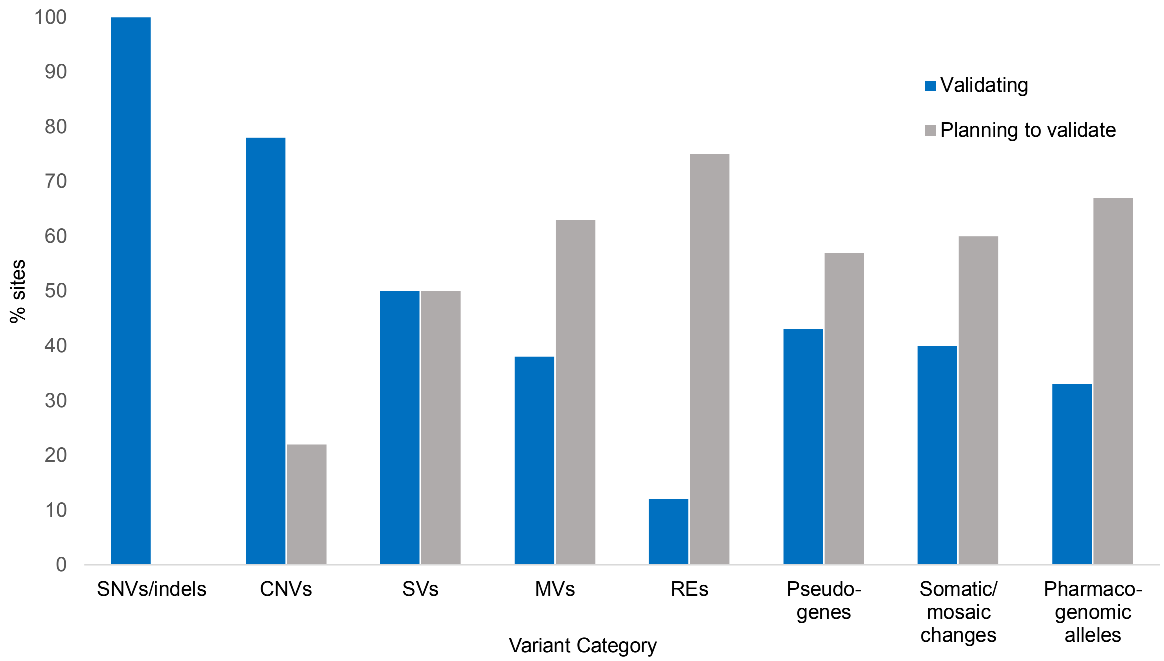

The percentage of Initiative sites that have either validated (blue) or plan to validate (grey) across the spectrum of variant types detectable by clinical whole-genome sequencing. SNV: single nucleotide variant; CNV: copy number variant; SV: structural variant; RE: repeat expansion

Supplementary Figure 2: Coverage titration for SNVs and Indel Accuracy

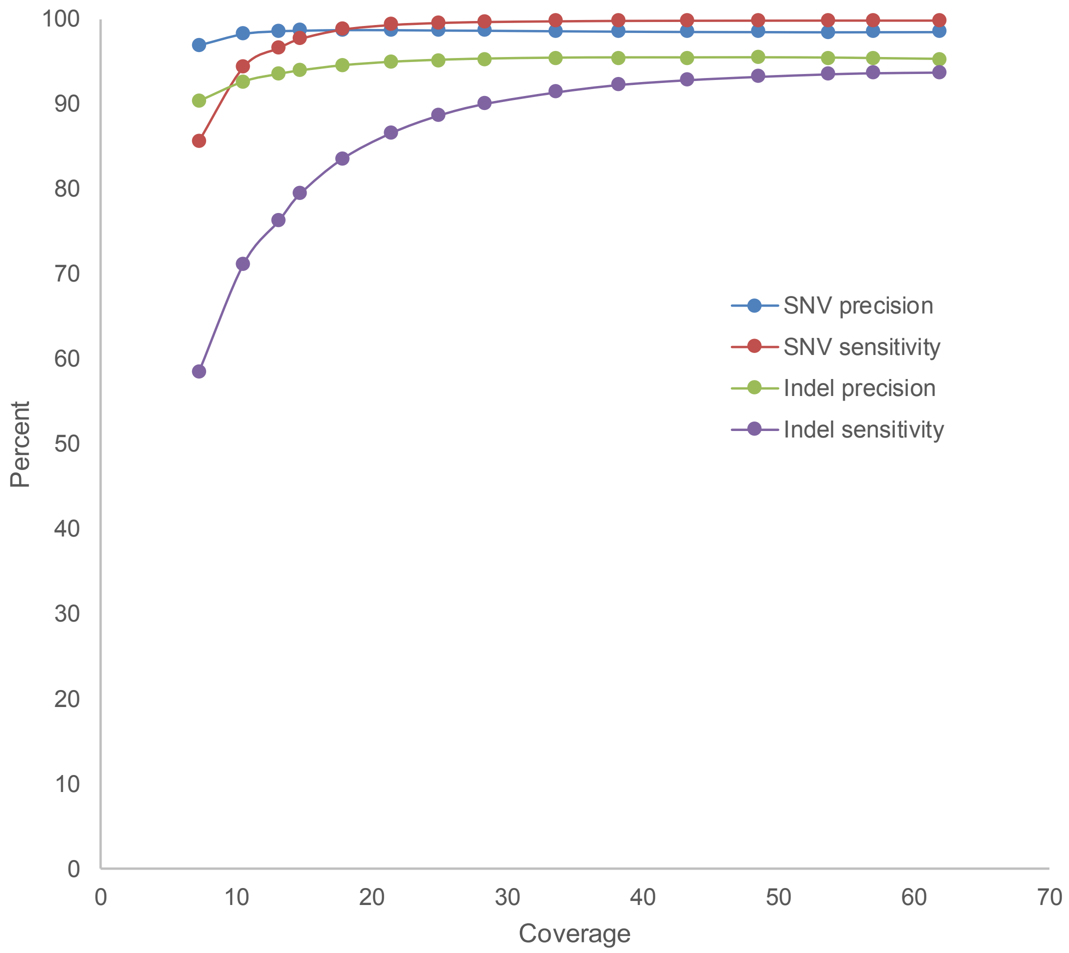

Representative coverage titration measuring SNV and indel precision using the Consensus Coding Sequence (CCDS) and the NA12878 Reference Standard truth set. Note that precision is equivalent to technical positive predictive value (TPPV).

Supplementary Figure 3: Fraction of callable bases in WGS versus WES

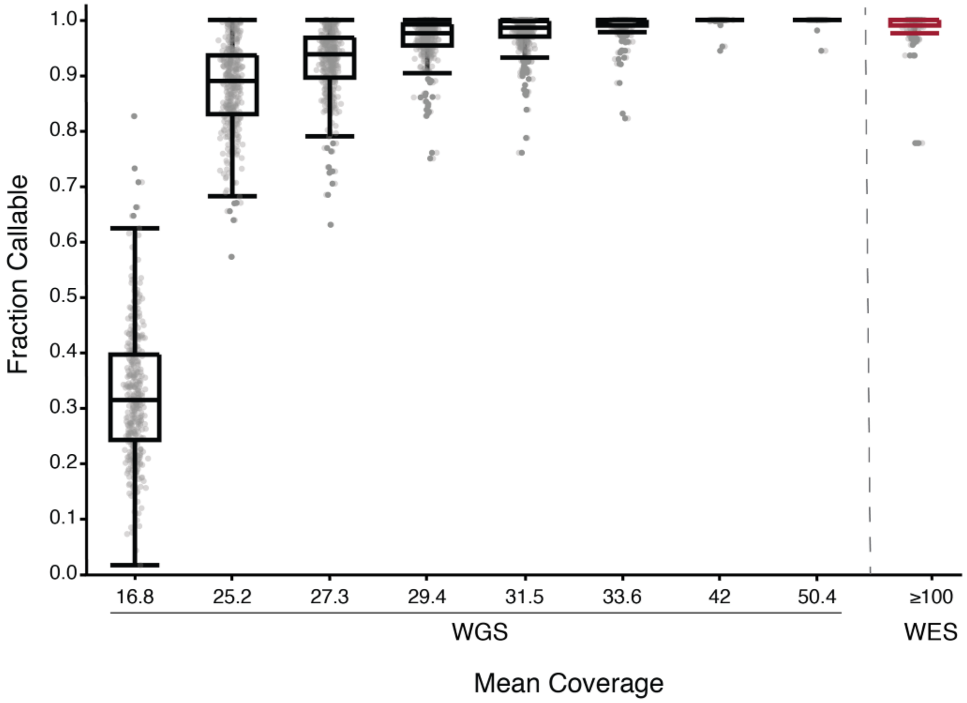

Fraction of callable bases in the ACMG secondary finding 56 genes at various whole-genome sequencing (WGS) mean coverages. A base within in the interval is considered 'callable' if it is covered at ≥20X, has a mapping quality ≥20, and a base quality of ≥20. The fraction of the interval that is callable is equivalent between WGS and whole-exome sequencing (WES) once genome mean coverage reaches >30X.

Supplementary Figure 4: Accuracy of indel calling across size ranges.

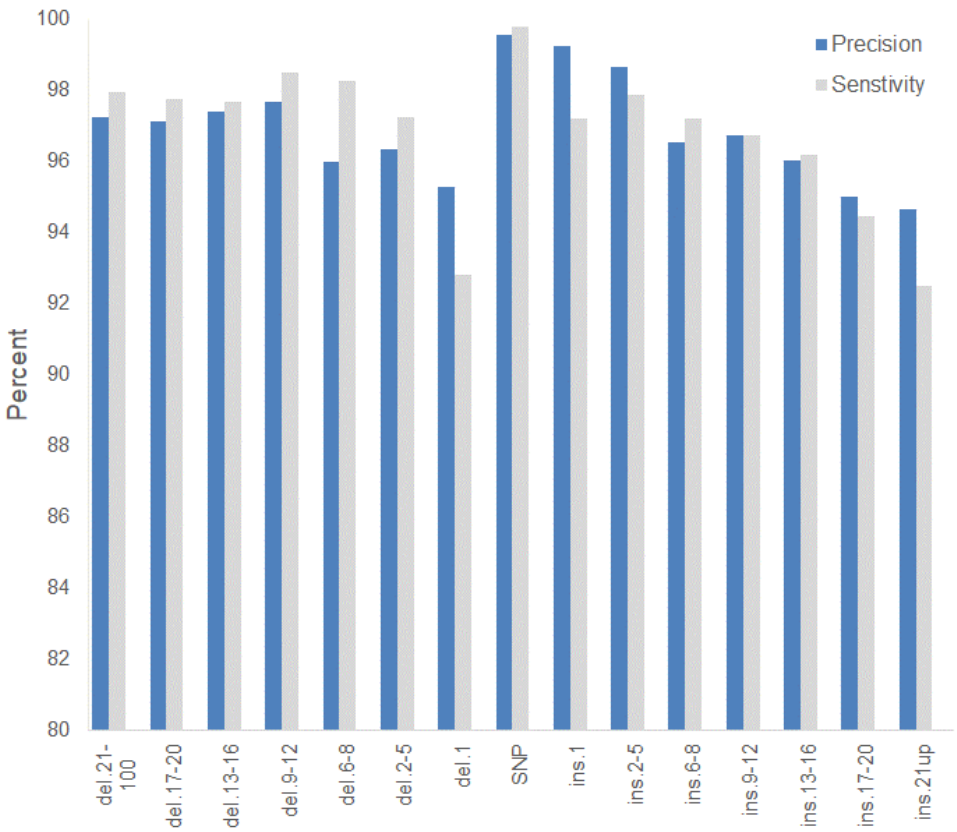

Representative data depicting the accuracy of indel calling across size ranges using the NA12878 Reference Standard truth set.

SUPPLEMENTARY TABLES

Supplementary Table 1: Accuracy Table for clinically relevant deletions (A) and duplications (B) called from whole-genome sequencing[1]

A. Deletions

		Comparator method			PPA[2]	NPA[3]	TPPV[4]
		Variant	Reference	Total			
CNV deletions >=10kb	Variant correct	72	5	77	1.00	0.998	0.9351
	Variant incorrect	-					
	Reference	-	2,374	2,374			
	total	72	2,378	2.450			

PPA: 95% Confidence Interval: 99.9% (94.9 to 99.9%)

B. Duplications

		Comparator method			PPA[2]	NPA[3]	TPPV[4]
		Variant	Reference	Total			
CNV duplications >=10kb	Variant correct	63	14	77	1.00	0.993	0.818
	Variant incorrect	-					
	Reference	-	2,099	2,099			
	total	63	2,113	2,176			

PPA: 95% Confidence Interval: 99.9% (95.13 to 99.9%)

Notes:
[1]Accuracy was determined using the comparator method outlined by the FDA.[17] The positive control dataset was constructed from 42 cases that underwent clinical chromosomal microarray analysis (CMA) at the SickKids Genome Diagnostics Laboratory. Briefly, positive controls were selected by surveying the most common microduplication and microdeletion syndromes with a reported frequency of >0.1% (22q11.2 Deletion Syndrome, 15q11.2 BP1 BP2 Deletion, 1p36 Deletion Syndrome, 16p11.2 Deletion Syndrome, Kleefstra Syndrome, 15q13.3 Deletion Syndrome, Phelan-McDermid Syndrome, Williams Syndrome, 16p12.2 Deletion Syndrome, 1q21.1 Duplication Syndrome, Koolen- De Vries Sydrome, Wolf-Hirschhorn Syndrome, 16p13.11 Deletion Syndrome, and 2q37 Deletion Syndrome). In addition, we choose additional cases with reportable variants >10kb in size and across a size spectrum. A total of 85 reportable deletions and 75 reportable duplications were used as the comparison truth set. All samples underwent genome sequencing with the Illumina HiseqX sequencing system and were analyzed for copy number variants using the read depth based calling in DRAGEN v.3.4.9.
[2]PPA: Positive Percent Agreement
[3]NPA: Negative Percent Agreement
[4]TPPV: Technical Positive Predictive Value

Supplementary Table 2: Assessment of Genome Completeness Across Laboratories

Medical Genome Initiative Laboratory	Total Reads[a]	Mean Coverage	Granular 3rd quartile	Granular median	Granular 1st quartile	% bases above 15
1	1,746,955,926	49.76	57	51	45	97.7
2	1,748,355,695	49.8	57	51	45	97.7
3	1,720,118,385	49	56	50	44	98.1

[a]Target is all coding exons +/-2bp as the region of interest with coverage set a variant calling cutoffs (MQ>17, BQ>10) (available by request)

Supplementary Table 3: Reference Standards and Positive Control Resource

		Variant Type								
		SNVs & Indels	CNVs (>10Kb)	SVs	Mitochondrial variants	Pseudogenes	REs	Somatic/ mosaic changes	Literature/Data	Source
Reference Samples	NA12878	100%[a]	40%	0	0	0	0	0	Zook et al[18] FTP Directory	NIST Reference Materials Link
Reference Samples	Other NIST standard (e.g. AJ/Asian trios)	71%	40%	50%	0	0	0	0	Zook et al[18] FTP Directory	NIST Reference Materials Link
Reference Samples	Platinum Genomes	29%	0	0	0	0	0	0	Eberle et al[8]	Platinum Genomes Link
Reference Samples	Venter/HuRef	14%	40%	0	0	0	0	0	Trost et al[1]	HuRef Link
Positive Controls	Disease specific positive controls[b]	86%	80%	50%	100%	100%	100%	50%	GeT-RM Link	GeT-RM Link
Positive Controls	Synthetic controls	0	0	0	33%	0	0	50%	Deveson et al[19]	Sequins Standards Link
Positive Controls	In silico data	0	20%	0	0	0	0	0	Escalona et al;[20] Duncavage et al[21]	
	No. positive control samples	10—85	7—42	>10	4—20	4—40	18—175	N/A		

[a]Percentage of Initiative sites using the specific reference or positive control sample per variant type
[b]Includes clinical sequencing/CMA controls and Coriell lines
SNV: single nucleotide variant; CNV: copy number variant; SV: structural variant; RE: repeat expansion

SUPPLEMENTARY DISCUSSION

TEST DEVELOPMENT AND OPTIMIZATION

There are several components of whole genome sequencing (WGS) test design that should be taken into consideration as part of test development and optimization, including sample and library preparation, sequencing methodology, sequence analysis, and annotation. These components generally follow current guidelines and are summarized below.

Sample preparation

Sample preparation for clinical WGS largely follow standard laboratory recommendations wherein proper labeling and sample tracking is critical to ensure the integrity of the final result. In the majority of cases, the laboratory may receive purified genomic DNA, whose quality meets or exceeds pre-defined parameters, or biological samples which include, but are not limited to, whole blood, blood spot on filter paper, saliva, buccal brush/swab, urinary sediment, tissue, or cultured cells, ensuring that there is sufficient sample for DNA preparation. Clinical WGS using short read technology is generally robust to DNA source but some specimen types, like saliva, have varying degrees of non-human DNA contamination that decrease overall genome coverage. In addition, the small amount of DNA needed (1ug) for PCR-free library preparation and followed by short read technology sequenced generally allows most specimen types of DNA sources to be used; however, it should be noted that some specimen types like blood spots may not yield enough DNA for PCR-free libraries and may limit downstream analysis (i.e. copy number or repeat expansion analysis). Moreover, it should be noted

that long-read sequencing technologies will have different DNA requirements and the laboratory may want to determine if the extraction technique is compatible. All members of this Initiative have validated clinical WGS with whole blood as a sample type, but only a few laboratories have validated alternate tissue sources like buccal, saliva, fibroblasts, and blood spots.

Library preparation

PCR-free library preparation is preferred for clinical WGS because it improves the evenness of coverage and other bioinformatics analyses such as calling copy number variants calling[1] and repeat-disease testing expansions.[2] However, when using DNA isolated from saliva samples, contamination of non-human (bacterial) sequence may be significant when using either a PCR or PCR-free library. Members of this initiative have observed bacterial contamination of between 8-30%, which affects overall coverage (unpublished data). When DNA quantity is limited, standardized amplification methods can be used for clinical WGS but limitations should be noted, including increased PCR duplicates.

Current sample preparation methods utilize sample indexing in which a sample-identifying oligonucleotide is introduced during the library preparation to allow for sample pooling. Even in cases where there is a 1:1 match between sequence yield from a single lane of a flowcell and the desired coverage of a single WGS sample, there are benefits to sample pooling. Pooling several samples and spreading the library across several lanes or instruments can offset the effects of any lane to lane or instrument to instrument variability. Similarly, if more sequence data is needed to reach coverage

targets, pooling many samples and sequencing can be a cost-effective option to top up coverage. Incorrect index assignment may be an issue with PCR-free libraries and compounded by choice of downstream sequencing technology; thus, using uniquely dual indexed libraries is recommended to mitigate index-hopping error. Pooling of libraries is common practice to achieve desired overall coverage but is a practical issue for laboratories to ensure that samples are balanced as there is no well-accepted solution for how best to quantify and normalize before loading onto the sequencers. Library quantification using quantitative PCR is standard across laboratories with some using low pass sequencing with a lower throughput sequencer to ensure final pooling is balanced.

Sequencers, Sequencing Methodology and Sequence Generation

There are several factors in the sequencing process that contribute directly to the analytical validity of the data being produced including the inherent accuracy and error rates of the sequencing chemistry and instrument detection method, inter- and intra-instrument variability, quantity and quality of sequence data produced per sample, mean coverage and evenness of coverage of the genome, alignment and mapping accuracy, and systematic bias or noise in the data. All of these factors contribute to the performance of downstream variant calling.

There are a handful of sequencing platforms on the market today, each with distinguishing features such as read length, error rates, and cost per base.[3,4] However, once throughput and cost are taken into consideration there are limited sequencing technology options for routine high throughput clinical WGS. The members of this

initiative are using both Illumina HiSeq™ and NovaSeq™ platforms for clinical WGS and all sites have either validated or are intending to validate the test on the NovaSeq™ 6000.

After sequencing, manufacturer-supplied software is used for demultiplexing and base calling and data is stored in fastq file format, which includes base quality scores. Base calling quality (expressed as a phred score) is an important measure of confidence of an individual call and is used in downstream variant calling.[5,6] Base quality scores can be influenced by systematic technical errors or biases introduced by the sequencing process itself. Some groups apply base quality score recalibration (BQSR) to apply machine-learning techniques to model and correct for these errors (https://software.broadinstitute.org/gatk/).

Alignment and Variant Calling

Alignment of reads to a reference genome is the most computationally intensive part of clinical WGS analysis, and there are both open source and commercial aligners available that differ in processing speeds. Although relatively standard, the laboratory should examine the effect of aligner not only on the accuracy of Single nucleotide variants (SNVs) and indel calls, but also other classes of genomic variation like structural variants (SVs) and copy number variants (CNVs). Choice of genome build (hg19 versus GRCh38) and alignment method (e.g. use of decoy sequences and alternative loci) can have large effects on the accuracy of more complex variant calls. Similarly, there are many variant callers that have been developed for the detection of specific types of genomic variation including SNVs, multinucleotide variants (MNVs;

variants that involve two or more adjacent nucleotide substitutions), indels, SVs, CNVs, repeat nucleotide expansions (REs), segments with high homology, and variations in the mitochondrial genome. Evaluation and deployment of several different algorithms may be necessary to achieve the appropriate calling accuracy. Additionally, the reliability and accuracy of variant callers for more complex variants (i.e. SVs) are not yet well established. The majority of participating laboratories in this Initiative are using either BWA/GATK or Illumina DRAGEN™ for genome alignment and small variant calling (SNVs and Indels).

Variant Annotation

Both curated and inferred information is attached to each variant. Different annotations may be used for the major types of allelic variation – SNVs, MNVs, indels, CNVs, repeat expansions, and mitochondrial variants. Information about the quality of the variant call (e.g. the count of the reads in which the variant appears) is carried forward from the variant calling program. A major goal of annotation is to harmonize the variant nomenclature so that variants observed in different individuals can be confidently compared and tabulated. This is relatively simple for small variant calls but becomes more of a challenge when trying to integrate complex genomic variants. The quality of each variant substitution in MNVs is tabulated independently, but the consequence of the adjacent substitutions must be considered together since the prediction for each by itself could be quite different than that inferred from the full sequence.

TEST VALIDATION

Clinical WGS requires a multi-faceted approach to analytical validation with some aspects that are unique to WGS setup and others that are relevant to analytical validation of laboratory tests in general. Aspects of test validation that are not necessarily unique to clinical WGS including sequencing bias, repeatability and reproducibility, limits of detection, interference, and regions of homology; these are highlighted below. We also discuss disease-specific variant class validation, software validation, and test modification and updates.

Sequencing Bias

Estimates of analytical performance can be subject to bias and it is important to identify potential sources of error in clinical WGS. Genome reference standards have become more robust and biases associated with their use have become less likely as both deep sequencing on multiple technical platforms with independent chemistries and inheritance information (e.g. through trios) are used to establish truth sets.[7,8] Bias in reference standards can be addressed during validation by judicious use of orthogonal testing methods. Sanger sequencing and CMA are generally used to confirm WGS variant and copy number results, respectively. It is important to note that the error rate for Sanger sequencing may be higher than NGS, and the resolution of CMA may be lower than WGS. Sequencing or data biases encountered by the authors included GC and AT rich sequence drop relative to sequencer performance, and systematic biases in variant calling associated with specific aligners and variant callers.

Repeatability and Reproducibility

The validation process should include any known potential technical sources of variation. This could include laboratory staff, flow cell technical replicates, instruments, consumables, and computational pipelines. As part of the validation process, it is recommended to have different technical scientists prepare libraries and examine within (repeatability) and between run consistency (reproducibility). The laboratory should specify concordance thresholds for repeatability and reproducibility required for diagnostic use prior to validation. The accepted thresholds will vary depending on variant type as one can expect to have much higher concordance for SNVs compared to CNVs where there is greater variability in calling accuracy for breakpoints. For clinical WGS, it is expected that small variant calling should exceed 99% concordance with 90% of the discordance resulting from no-calls and no more than 10% from discordant genotype calls. These studies do not require gold standard reference materials; technical replicates can be used to calculate pair-wise positive agreement or pair-wise negative agreement.

Limits of detection

Germline mosaic and somatic variants relevant to non-neoplastic diseases can be detected with clinical WGS.[9,10] Given the decreased depth of coverage that is feasible for WGS compared to exome and panel testing, members of this Initiative have noted limited sensitivity for small variants whereas larger mosaic CNVs are easier to detect.[11] Laboratories reporting mosaicism should define the limits of detection for each variant type during validation and this will be tied to coverage targets. While mixing two pure

samples at variable percentages is an accepted method to establish mosaicism sensitivity, some patient samples must be used. The limit of detection for clinical WGS should be validated in a defined specimen type such as a blood sample or tissue biopsy.

Interference

The laboratory must identify and document any interfering substances that might occur in routine clinical samples thereby reducing the quality of the overall sequence. One example is saliva specimens, which contain varying amounts of bacterial genome contamination. High levels of contamination will reduce the number of reads that map to the human reference sequence and will thus reduce usable coverage and as well as the quality and number of variant calls.[12] There are no laboratories in this initiative that currently use saliva as a tissue source for clinical WGS due to this contamination issue. Several are planning to establish performance criteria that will allow for saliva as a valid biospecimen type.

Regions of Homology

Clinical WGS produces sequence reads from all parts of the genome. The ability to unambiguously align and map reads to the reference genome is reduced in regions of sequence similarity such as low copy repeats, paralogous genes, pseudogenes and simple repeats. These can be stratified during validation to assess calling accuracy. Regions of homology reduce the sensitivity of clinical WGS by making it difficult or impossible to reliably call variants in some genes. Pseudogenes are particularly

problematic as they typically harbor variants that would be deleterious to function if they occurred in the active copy of a gene. As part of a validation process, a laboratory should identify all clinically relevant areas with these technical issues that are excluded from the WGS test definition and make this list available for upon request.

Disease-Specific Variant Validation

Although our test definition for WGS is broad and agnostic to phenotype, there are some medically relevant diseases in regions of homology can be assessed and reported. It is now feasible to use clinical WGS data combined with specialized algorithms to provide a definitive genotype for these conditions or signal that there is an abnormality that could be resolved by orthologous testing.[13] Laboratories validating clinical WGS should obtain appropriate samples representing patients with these disorders and demonstrate the ability to detect the specific abnormality. For laboratories that choose to implement a customized variant caller to address a particular region [e.g. SMN1 and SMN2 associated with Spinal Muscular Atrophy (SMA)], this group recommends a dedicated validation effort employing >50 disease positive samples representing the spectrum of disease-relevant alleles for development and validation. Depending upon the performance of the caller, orthogonal confirmation of findings before reporting may be necessary in some cases.

Software Validation

Validation studies should document all the computational components needed to compile, install, and run the specialized clinical WGS bioinformatics pipelines.[14] A

system of version control and detailed documentation of code changes is necessary for any deployment of software updates. Validation is performed on a defined version of the software and data analysis should be repeated each time software components and reference data files are updated. Laboratories should have a clear definition of major versus minor updates that should determine the level of validation. When software components are changed, the testing mechanism must revalidate local test performance and the effect of those changes on the clinical WGS variant calling and annotation process. This is sometimes called “deep testing” to indicate that an effort is made to identify errors or altered test performance elsewhere in the process that may arise when an isolated process is improved. Ideally, software validation should include processes for continuous integration of upgrades and improvements. This may require that software testing is to some degree automated when incremental changes are introduced. It is crucial that new failures are detected efficiently, and the underlying problems identified and addressed.

Test Modification and Updates

Periodic updating or modifying a clinical WGS test is imperative due to the continual development and advancement of all the methodological components available for WGS. After initial clinical WGS test validation, the laboratory may need to make changes to the wet bench process and bioinformatics pipeline in order to increase efficiency, improve accuracy, expand reporting of variant classes and ensure that the highest quality results are obtained.

Modifications to the validated clinical WGS test may include minor or major changes. Laboratories should have a strategy in place for these updates that includes a careful re-evaluation of previously analyzed datasets or re-calculation of the performance specifications. Minor changes such as instrument software updates and updating a version of the tools/algorithm(s) used in the pipeline require running previously analyzed cases/data sets to ensure there are no inadvertent issues with the updates. Major changes such as validating a new instrument (sequencer), additional sample type, library preparation method (PCR vs. PCR free), or human genome build require a recalculation of sensitivity and precision and a reassessment of the types of variants detected.

QUALITY MANAGMENT

Quality control and quality assurance are important components of a quality management program following applicable guidelines from CLIA (www.cdc.gov/clia), CAP (https://www.cap.org/), and ISO (www.iso.org). Below we expand on the sequencing quality and performance metrics section from the main text and add more detail on quality management from alignment, variant calling and annotation.

Alignment and Variant Calling Metrics

After de-multiplexing, it is expected that only a small fraction of reads will fail to be assigned to an expected sample. In alignment, mapping quality scores are assigned and recorded in the output files, which can have a variety of coding and compression formats including SAM, BAM, and CRAM. In variant calling, metrics are associated with

each variant as well as global metrics for all variants identified. For example, single base variant call metrics typically include variant call quality score, total depth of coverage at the variant position, variant allele fraction (number and percentage of reads with the variant reported), predicted zygosity, and strand bias for paired-end sequencing. Global variant metrics often captured include percent of different variant types [e.g., heterozygous calls vs homozygous calls, indels vs SNVs, or variant types (i.e. nonsense variants, silent variants, missense variants)], portion and ratios of base substitutions [transition/transversion (Ti/Tv)], percent of novel variants as compared to a standard reference (dbSNP, gnomAD, etc), and concordance rates with reference variant/sequence, as applicable.

Additional quality control metrics also make use of the alignment and variant calling data, in order to detect contamination and to establish sample identity. Genome sequencing can identify instances of contamination from exogenous species (often bacterial or viral species) and patient samples. Contamination thresholds should be set (typically <2% cutoff) and should be substantially lower than reported minimum detection level used for somatic variant and mosaic variant detection.

Because of the numerous steps required in WGS, there is the opportunity for sample or data misidentification between accessioning and reporting. This is especially relevant for the laboratories that do not require orthogonal confirmation of reportable variants. In this instance, the laboratory can identify and utilize markers present in the genome data and compare them to an orthogonal assay to confirm that the report corresponds to the correct individual, although this will not mitigate against pre-analytical sample labeling errors.

Variant quality scores are generated for each position including those that are apparently homozygous for the reference allele. In general, a laboratory produces a vcf file containing only those positions that differ from reference. A much larger file with scores for all positions called a gvcf can also be generated. Variant quality metrics are central to the process because errors in variant calling will negatively affect annotation, filtering, classification, interpretation and reporting steps. Variant calling metrics are used to estimate the accuracy of a particular genotype call. Through robust validation, the laboratory must establish the acceptable variant quality thresholds that provide confidence that the variant is a true positive (e.g. variant allele fraction). In addition, the genomic context should be considered since variants within difficult to sequence regions or those with low mapping quality (e.g. repetitive DNA, SINES, segmental duplications) are prone to false positive calls.[15] It is common practice to confirm all variants using an orthogonal method prior to reporting, however as laboratories gain more experience with clinical WGS and collect data regarding variant confirmation rates and test accuracy, orthogonal confirmation of high confidence variants passing established quality thresholds will no longer be required.[16]

Variant Annotation

Variant annotation names each variant using standardized nomenclature and links it with information from various databases and the medical literature. The Human Genome Variation Society (HGVS) and International Standing Committee on Human Cytogenetic Nomenclature (ISCN) nomenclature guidelines are currently widely adopted by laboratories, databases, and the medical literature. Although these

guidelines generally address the variety of variant types detected by WGS the nomenclature is becoming increasingly cumbersome to use and thus a more flexible reference based variant naming may be a solution.

Proper annotation of variants is necessary for downstream variant filtering, prioritization, classification, and interpretation. Given the significance of variant annotation, careful evaluation of software and other tools should be carried out to eliminate potential sources of error, such as incorrect application of HGVS/ISCN nomenclature guidelines or incorrect matching of database values to called variants. Currently, there are no consensus standards for variant annotation and therefore no agreed upon acceptance criteria for validation and implementation of variation annotation. Standards in annotation are being developed by the GA4GH variant annotation working group (https://ga4gh-gks.github.io/variant_annotation.html) through the Genomic Knowledge Standards (GKS) Work Stream (https://ga4gh-gks.github.io/).

Validation of variant annotation tools and annotation sources should focus on evaluating performance such that a uniform and consistent set of annotations are routinely produced by the software and that any differences between expected and observed annotations are systematic, explainable and documented. For clinical WGS, a thorough evaluation of software performance is recommended, as some variant types that may be evaluated and reported as part of a clinical WGS assay, including noncoding variants, CNVs, and other SVs, are now routinely identified by most NGS-based tests. These variant types may be at greatest risk for errors with regard to correct application of HGVS/ISCN nomenclature and the ability to correlate variant identity with appropriate databases.

SUPPLEMENTARY REFERENCES

1. Trost B, Walker S, Wang Z, et al. A Comprehensive Workflow for Read Depth-Based Identification of Copy-Number Variation from Whole-Genome Sequence Data. *Am J Hum Genet.* 2018;102(1):142-155.
2. Dolzhenko E, van Vugt J, Shaw RJ, et al. Detection of long repeat expansions from PCR-free whole-genome sequence data. *Genome Res.* 2017;27(11):1895-1903.
3. Mardis ER. DNA sequencing technologies: 2006-2016. *Nat Protoc.* 2017;12(2):213-218.
4. Goodwin S, McPherson JD, McCombie WR. Coming of age: ten years of next-generation sequencing technologies. *Nat Rev Genet.* 2016;17(6):333-351.
5. Ewing B, Hillier L, Wendl MC, Green P. Base-calling of automated sequencer traces using phred. I. Accuracy assessment. *Genome Res.* 1998;8(3):175-185.
6. Ewing B, Green P. Base-calling of automated sequencer traces using phred. II. Error probabilities. *Genome Res.* 1998;8(3):186-194.
7. Zook JM, Catoe D, McDaniel J, et al. Extensive sequencing of seven human genomes to characterize benchmark reference materials. *Sci Data.* 2016;3:160025.
8. Eberle MA, Fritzilas E, Krusche P, et al. A reference data set of 5.4 million phased human variants validated by genetic inheritance from sequencing a three-generation 17-member pedigree. *Genome Res.* 2017;27(1):157-164.
9. Dou Y, Kwon M, Rodin RE, et al. Accurate detection of mosaic variants in sequencing data without matched controls. *Nature biotechnology.* 2020;38(3):314-319.
10. Yuen RK, Thiruvahindrapuram B, Merico D, et al. Whole-genome sequencing of quartet families with autism spectrum disorder. *Nat Med.* 2015;21(2):185-191.
11. Scocchia A, Wigby KM, Masser-Frye D, et al. Clinical whole genome sequencing as a first-tier test at a resource-limited dysmorphology clinic in Mexico. *NPJ Genom Med.* 2019;4:5.
12. Mandelker D, Schmidt RJ, Ankala A, et al. Navigating highly homologous genes in a molecular diagnostic setting: a resource for clinical next-generation sequencing. *Genet Med.* 2016;18(12):1282-1289.
13. Chen X, Sanchis-Juan A, French CE, et al. Spinal muscular atrophy diagnosis and carrier screening from genome sequencing data. *Genet Med.* 2020.
14. Roy S, Coldren C, Karunamurthy A, et al. Standards and Guidelines for Validating Next-Generation Sequencing Bioinformatics Pipelines: A Joint Recommendation of the Association for Molecular Pathology and the College of American Pathologists. *J Mol Diagn.* 2018;20(1):4-27.
15. Goldfeder RL, Priest JR, Zook JM, et al. Medical implications of technical accuracy in genome sequencing. *Genome Med.* 2016;8(1):24.
16. Lincoln SE, Truty R, Lin CF, et al. A Rigorous Interlaboratory Examination of the Need to Confirm Next-Generation Sequencing-Detected Variants with an Orthogonal Method in Clinical Genetic Testing. *J Mol Diagn.* 2019;21(2):318-329.
17. FDA. Considerations for Design, Developmment, and Analytical Validation of Next Generation Sequencing (NGS) - Based In Vitro Diagnostics (IVDs) Intended to Aid in the Diagnosis of Suspected Germline Diseases. In: Administration UFaD, ed2018.
18. Zook JM, McDaniel J, Olson ND, et al. An open resource for accurately benchmarking small variant and reference calls. *Nature biotechnology.* 2019;37(5):561-566.

19. Deveson IW, Chen WY, Wong T, et al. Representing genetic variation with synthetic DNA standards. *Nat Methods.* 2016;13(9):784-791.
20. Escalona M, Rocha S, Posada D. A comparison of tools for the simulation of genomic next-generation sequencing data. *Nat Rev Genet.* 2016;17(8):459-469.
21. Duncavage EJ, Abel HJ, Merker JD, et al. A Model Study of In Silico Proficiency Testing for Clinical Next-Generation Sequencing. *Arch Pathol Lab Med.* 2016;140(10):1085-1091.
